# TCR-Engineered T Cells Meet New Challenges to Treat Solid Tumors: Choice of Antigen, T Cell Fitness, and Sensitization of Tumor Milieu

**DOI:** 10.3389/fimmu.2013.00363

**Published:** 2013-11-08

**Authors:** Andre Kunert, Trudy Straetemans, Coen Govers, Cor Lamers, Ron Mathijssen, Stefan Sleijfer, Reno Debets

**Affiliations:** ^1^Laboratory of Experimental Tumor Immunology, Erasmus MC Cancer Institute, Rotterdam, Netherlands; ^2^Department of Medical Oncology, Erasmus MC Cancer Institute, Rotterdam, Netherlands

**Keywords:** antigens, inhibitory micro-milieu, solid tumors, T cell avidity, T cell co-stimulation, T cells, TCR affinity, TCR transgenes

## Abstract

Adoptive transfer of T cells gene-engineered with antigen-specific T cell receptors (TCRs) has proven its feasibility and therapeutic potential in the treatment of malignant tumors. To ensure further clinical development of TCR gene therapy, it is necessary to target immunogenic epitopes that are related to oncogenesis and selectively expressed by tumor tissue, and implement strategies that result in optimal T cell fitness. In addition, in particular for the treatment of solid tumors, it is equally necessary to include strategies that counteract the immune-suppressive nature of the tumor micro-environment. Here, we will provide an overview of the current status of TCR gene therapy, and redefine the following three challenges of improvement: “choice of target antigen”; “fitness of T cells”; and “sensitization of tumor milieu.” We will categorize and discuss potential strategies to address each of these challenges, and argue that advancement of clinical TCR gene therapy critically depends on developments toward each of the three challenges.

## TCR Gene Therapy: Clinical Potency and Toxicities

T cells possess distinct properties such as the ability to specifically recognize tumor antigens, serially kill tumor cells, self-replicate, form memory and induce a complete tumor response. It is because of these properties that the therapeutic use of T cells in certain types of cancer may be advantageous when compared to drugs, antibodies, or small molecule inhibitors.

T cell therapy intends to treat cancer by transferring autologous and *ex vivo* expanded T cells to patients. Therapy with tumor-infiltrating T lymphocytes (TILs) preceded by non-myeloablative lymphodepletion resulted in objective responses in about 50% of metastatic melanoma patients in two different medical centers (1, 2). Equally notable were the durable complete responses observed in these trials that ranged between 10 and 22% (ongoing for more than 3 years) (1, 2). Likewise, adoptive transfer of tumor-specific T cell clones generated from autologous peripheral T cells resulted in regression of individual metastases, and responses in 8 out of 10 melanoma patients (3). In addition, co-culture of peripheral T cells with artificial antigen-presenting cells (APC) loaded with tumor antigens resulted in T cells that were clinically effective in four out of seven evaluable melanoma patients (4). Response rates observed with T cell therapy are generally higher than those observed for other treatments of melanoma, such as chemotherapeutic drugs, high-dose cytokines, inhibitors of kinases, or antibodies against T cell co-inhibitory molecules. See Table 1 for an overview of clinical outcomes of T cell therapies and other treatments of melanoma.

**Table 1:** **Overview of standard and experimental none-gene-based therapies for metastatic melanoma**.

Therapy	Function	Type of trial	OR (%)^a^	CR (%)^a^	Reference
**T CELL THERAPY**					
Tumor-infiltrating lymphocytes (TILs)	Adoptive transfer of tumor-specific T cells	n.c.	52/93 **(56)**	20/93 **(22)**	(1)
		n.c.	15/31 **(48)**	3/31 **(10)**	(2)^b^
T cell clones		n.c.	8/10 **(80)**	n.r.	(3)
“Educated T cells”		n.c.	4/9 **(44)**	1/9 **(11)**	(4)
**STANDARD THERAPY**					
High-dose IL-2	Cytokine that induces T cell growth	n.c.	43/270 **(16)**	16/270 **(6)**	(178)
Dacarbazine (DTIC)	Drug that alkylates DNA	Phase III trial	18/149 **(12)**	4/149 **(3)**	(179)
Vemurafenib (PLX-4032)	Small molecule that inhibits BRAF kinase activity	Phase III trial	106/219 **(48)**	2/219 **(1)**	(180)
**EXPERIMENTAL THERAPY**					
Dabrafenib	Small molecule that blocks BRAF kinase activity	Phase III trial	29/54 **(54)**	n.r.	(181)
Dabrafenib + Trametinib	Small molecules that block BRAF and MEK kinase activities	Phase III trial	41/54 **(76)**	n.r.	(181)
Ipilimumab (MDX-010) + vaccination	Antibody that blocks T cell CTLA4	Phase III trial	39/137 **(28)**	3/137 **(2)**	(182)
Ipilimumab + DTIC		Phase III trial	34/252 **(14)**	26/252 **(10)**	(183)
Nivolumab (MDX-1106)^c^	Antibody that blocks T cell PD1	Phase I trial	5/39 **(13)**	1/39 **(3)**	(184)
		Phase I trial	26/94 **(28)**	n.r.	(185)
Nivolumab + Ipilimumab		Phase I trial	21/53 **(40)**	n.r.	(186)
Lambrolizumab (MK-3475)	Antibody that blocks T cell PD1	Phase I trial	51/135 **(38)**	n.r.	(187)
Anti-PD-L1 (MDX-1105)	Antibody that blocks tumor cell PDL1	Phase I trial	17/135 **(13)**	n.r.	(188)

*^a^ OR, objective responses; CR, complete responses; both according to Response Evaluation Criteria for Solid Tumors (RECIST). Number of patients with responses = before dash; total number of patients treated = after dash; percentage of responses = between brackets*.

*^b^ Dr. Jacob Schachter, Cellular Therapy of Cancer Symposium, September 24–27th, Montpellier, France, 2010*.

*^c^ This study included patients with metastatic melanoma, but also patients with renal cell carcinoma, colorectal cancer, prostate cancer, and non-small-cell lung cancer*.

*BRAF, gene responsible for production of B-Raf-kinase; CTLA4, cytotoxic T-lymphocyte antigen 4; IL-2, Interleukin 2; n.c., not classified; n.r., none reported; mAb, monoclonal antibody; MAPK, mitogen-activated protein kinase; PD1, programed cell death 1 receptor; PDL1, programed cell death 1 ligand*.

**Figure 1:**
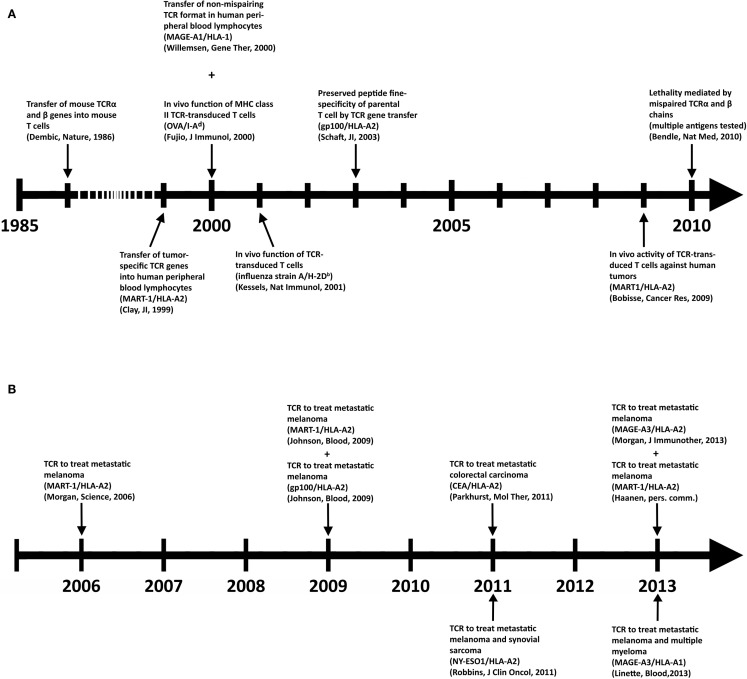
**Key achievements in the field of TCR gene therapy directed against solid tumors**. **(A)** Timeline of selected preclinical findings that have contributed to the development of TCR gene therapy. **(B)** Timeline of clinical findings with TCR gene-engineered T cells. Details with respect to clinically used TCRs can be found in Table 2.

Despite its clinical successes, T cell therapy has its limitations in availability and generation of therapeutic T cells for a larger group of patients. Genetic introduction of T cell receptors (TCRs) or chimeric antigen receptors (CARs) into autologous T cells, termed gene-engineering of T cells, can provide an alternative that is more widely applicable and can potentially be extended to multiple types of cancer (5). Key preclinical achievements and clinical tests with TCR-engineered T cells, the focus of the current review, are depicted in Figures 1A,B, respectively. Therapeutic advances with CAR-engineered T cells is reviewed elsewhere (6). The principle of clinical TCR gene therapy is straightforward: transferral of TCRαβ genes into T cells; *ex vivo* expansion of T cells; and infusion of T cells into the patient. In this way, TCRα and β genes are used as “off the shelf” reagents to confer tumor reactivity to patients whose tumor expresses the appropriate antigen and HLA restriction element. At the moment of writing this review, eight clinical trials using TCR-engineered T cells have reported their results (see Figure 1B and Table 2 for details), and at least another 10 trials using TCR-engineered T cells are open and actively recruiting patients or will recruit patients soon^1^.

**Table 2:** **T cell receptor gene therapy trials – an update on efficacy and safety**.

Target antigen (epitope)	Original T cell clone/lines	Tumor type	OR (%)	CR (%)	Toxicity (%)^a^	Type of toxicity	Reference
MART-1(AAG)/HLA-A2	TIL clone DMF4 from responding patient	Metastatic melanoma	2/17 **(12)**	n.r.	0/17 **(0)**	n.r.	(189)
MART-1(AAG)/HLA-A2	TIL clone DMF5 from responding patient with high *in vitro* avidity	Metastatic melanoma	6/20 **(30)**	n.r.	9/36 **(25)**	Severe melanocyte destruction in skin, eye, and ear (in some cases leading to uveitis and hearing loss)	(190)
gp100(KTW)/HLA-A2	Splenocytes from immunized mouse	Metastatic melanoma	3/16 **(19)**	n.r.	
CEA(IMI)/HLA-A2	Splenocytes from immunized mouse; TCR is affinity-enhanced	Metastatic colorectal carcinoma	1/3 **(33)**	n.r.	(3/3) **(100)**	Severe inflammation of colon	(191)
NY-ESO1(SLL)/HLA-A2	T cell clone 1G4 from human subject; TCR is affinity-enhanced	Metastatic melanoma	5/11 **(45)**	2/11 **(18)**	0/11 **(0)**	n.r.	(192)
		Metastatic synovial sarcoma	4/6 **(67)**	0/6 **(0)**	0/6 **(0)**	
MAGE-A3(KVA)/HLA-A2	Splenocytes from immunized mouse; TCR is affinity-enhanced	Metastatic melanoma	5/9 **(55)**	2/9 **(22)**	3/9 **(33)**	Changes in mental status, two patients fell into coma and subsequently died, one patient recovered	(29)
MART-1(ELA)/HLA-A2	T cell clone 1D3 from human subject; TCR is codon-optimized and murinized	Metastatic melanoma	n.r.	n.r.	1/1 **(100)**	Lethal cardiac toxicity in one patient	^b^
MAGE-A3(EVD)/HLA-A1	T cell clone a3a from human subject; TCR is affinity-enhanced	Metastatic melanoma and multiple myeloma	n.r.	n.r.	2/2 **(100)**	Lethal cardiac toxicity in two patients	(30)

*OR, objective responses; CR, complete responses, both according to Response Evaluation Criteria for Solid Tumors (RECIST). Number of patients with responses = before dash; total number of patients = after dash; percentage of responses = between brackets*.

*^a^ Number of patients with Serious Adverse Events (toxicity grading ≥3 according to National Cancer Institute common toxicity criteria) and total number of patients treated are put before and after dash, respectively*.

*^b^ Dr. John Haanen, Cellular Therapy of Cancer Symposium, London, UK, February 27th–March 2nd, 2013*.

*CEA, carcinoembryonic antigen; gp, glycoprotein; HLA, human leukocyte antigen; MAGE, melanoma-associated antigen; MART, melanoma antigen recognized by T cells; n.r., none reported; NY-ESO1, New York esophageal squamous cell carcinoma 1*.

Most clinical TCRs tested so far were HLA-A2-restricted and directed against either melanoma-associated antigen recognized by T cells 1 (MART-1), glycoprotein (gp) 100, carcinoembryonic antigen (CEA), p53, melanoma-associated antigen (MAGE-)A3, or New York esophageal squamous cell carcinoma antigen (NY-ESO)1. Another TCR tested clinically was HLA-A1-restricted and directed against MAGE-A3. Collectively, these trials have not only demonstrated feasibility but also demonstrated significant clinical responses in patients with metastatic melanoma, colorectal carcinoma, and synovial sarcoma (Table 2). Responses, although variable and tested in a cumulative number of about 80 patients (based on trials listed in Table 2), ranged from 12 to 67%. Notably, the finding that TCR gene-engineered T cells were able to traffic to the central nervous system and cause complete responses of brain metastasis in patients with melanoma was not only encouraging but also underscored the strength of T cell therapy toward metastasized and poorly accessible tumors (7). Clinical testing, however, also clearly demonstrated that therapy is currently hampered by treatment-related toxicity and a transient nature of tumor regression. Treatment-related toxicity became evident from studies with TCRs, in particular those of high-affinity, directed against antigens that are over-expressed on tumors but also expressed on healthy cells. Toxicities included severe but treatable inflammation of skin, eyes, ears (MART-1/HLA-A2; gp100/HLA-A2), and colon (CEA/HLA-A2). In addition, lethal neurological toxicities were observed in two patients when targeting MAGE-A3/HLA-A2, and lethal cardiac toxicities were observed in three patients when targeting MART-1/HLA-A2 (another epitope as above) or MAGE-A3/HLA-A1. The transient nature of tumor regression became evident from observations that anti-tumor responses are initially significant but not sustainable and ultimately incomplete in 80–90% of patients. Table 2 offers an up-to-date and detailed overview of toxicities as well as clinical responses reported for TCR gene therapy trials.

**Figure 2:**
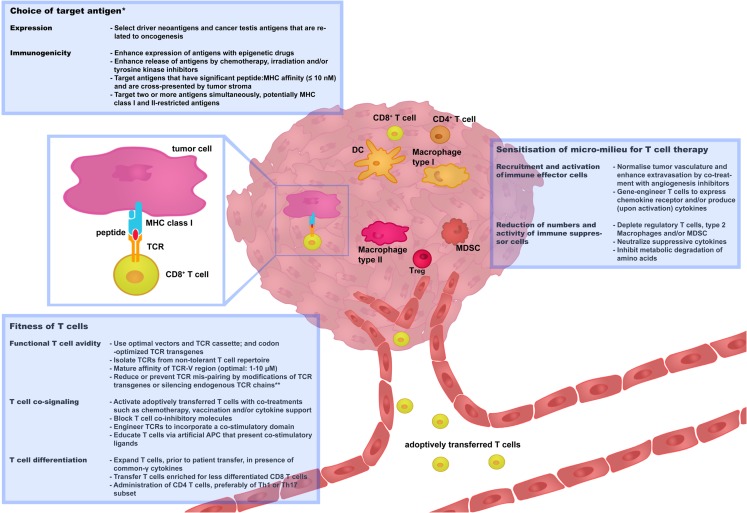
**Three challenges that determine the success rate of TCR gene therapy**. In this figure, recent and successful strategies to improve TCR gene therapy have been categorized along three renewed challenges: “choice of target antigen”; “fitness of T cells”; and “sensitization of micro-milieu for T cell therapy.” Boxes provide selected strategies that are discussed in more detail in Sections “Choice of Target Antigen,” “Fitness of T cells,” and “Sensitization of Micro-Milieu for T Cell Therapy.” We propose that advancement of clinical TCR gene therapy is guided by the principles of these challenges. *Independent of choice of target antigen, it is recommended to perform stringent *in silico* analysis and preclinical tests to confirm that healthy cells do not express the target antigen prior to proceeding with the clinical testing of TCR-engineered T cells. **Strategies to reduce or prevent TCR mis-pairing do not only enhance T cell avidity but also reduce the potential risk of off-target toxicity. APC, antigen-presenting cells; DC, Dendritic cells; MDSC, myeloid-derived suppressor cells; Th, T helper cells; Treg, T regulatory cells.

Strategies that aim at preventing or limiting toxicities as well as tumor recurrences have already been developed, some of which need further preclinical testing and some of which have already been implemented in clinical trials. In this review, we have categorized these strategies along three renewed challenges, i.e., “choice of target antigen”; “fitness of T cells,” and “sensitization of micro-milieu for T cell therapy,” as illustrated in Figure 2. We propose and will argue that optimizations along each or combinations of these challenges will contribute most significantly to the advancement of clinical TCR gene therapy.

## Choice of Target Antigen

Ideally, target antigens are selectively expressed by tumor tissue and not healthy tissue, and hence not expected to evoke a response against self. At the same time, target antigens should have proficient immunogenicity to initiate an effective anti-tumor response.

### Selective expression

Tumor-associated antigens (TAAs) can generally be divided into four groups (8).

- Differentiation antigens: cell surface proteins that are expressed at different stages of tissue development or cell activation. Expression of these antigens may discriminate tumor cells from surrounding healthy cells, but expression by healthy cells is not absent. Examples include MART-1, gp100, CEA, and tyrosinase related protein (TRP)1 and 2.
- Over-expressed antigens: cell surface proteins that are highly, but not selectively, expressed by tumor cells when compared to healthy cells. Examples include the epidermal growth factor receptor (HER)2 or survivin.
- Cancer Testis Antigens (CTAs): proteins that are expressed by tumors and a limited number of healthy and adult cell types. A defined number of CTAs may not be expressed by healthy adult cell types. Examples include MAGE-A1, MAGE-C2, and NY-ESO1.
- Neo-antigens: proteins that result from gene mutations or aberrations in tumor cells. These proteins are uniquely expressed by tumor cells but not healthy cells. Examples include mutated protein (p)53, B-Raf kinase, and cyclin-dependent kinase 4 (CDK4).

Looking at these four groups of TAAs, CTAs, and neo-antigens may represent the best available choices for therapy with TCR-engineered T cells. With respect to CTAs, over several hundreds of genes have been identified (see for a full description of CTAs^2^). Approximately 40 of these genes belong to multigene families that are located on the X-chromosome. A selected number of mostly X-chromosome-located CTAs may be of interest for T cell therapy. *First*, these antigens are not expressed by healthy tissues except testes and placentas (determined using RT-PCR), and these latter tissues do not express Major Histocompatibility (MHC) molecules and cannot be targeted by T cells (9). *Second*, CTAs are expressed by tumor tissues of various histological origins as a result of aberrant epigenetic regulation (9), and expression of CTAs has been associated with advanced stages of disease and unfavorable patient prognosis (10). Along these lines, there is evidence that MAGE proteins are related to oncogenesis as they suppress p53-dependent apoptosis and cause fibronectin-controlled increase in tumor cell proliferation and metastasis (11–15). *Third*, CTAs are immunogenic proteins that have been reported to induce both humoral and cell-mediated immune responses in patients without the concomitant induction of toxicities (10, 16, 17). Undeniably, current patient studies emphasize the need for careful identification of target CTAs. In one study, Robbins and colleagues demonstrated that a TCR directed against NY-ESO1/HLA-A2 showed significant anti-tumor responses in patients with metastatic melanoma and synovial sarcoma without detectable toxicities (Table 2). Unexpectedly, in another study using a TCR directed against MAGE-A3/HLA-A2, two patients with metastatic melanoma lapsed into coma and died. These adverse events were most likely caused by T cell recognition of rare neurons that were positive for MAGE-A12 and possibly MAGE-A9 antigens, which contain shared or highly similar epitopes compared to MAGE-A3 antigen (Table 2). In a third study, in which a TCR was used directed against MAGE-A3/HLA-A1, one patient with melanoma and one patient with myeloma suffered from cardiovascular toxicity and died. This toxicity was possibly caused by T cell recognition of a similar but not identical peptide from the muscle protein titin (so-called “off-target” toxicity, Table 2).

With respect to neo-antigens, the expression of these antigens may vary significantly among different patients, but their expression is unique to tumor tissues. In case a neo-antigen is the result of “driver mutations,” the antigen may constitute an ideal target for T cell therapy. Driver mutations are related to oncogenesis, may be linked to known genes (∼400), and may provide tumors with a selective growth advantage (18, 19). Nevertheless, it is important to realize that only 15% of up to 100,000 mutations that are encountered in tumor genomes are considered “driver” mutations (18, 20). Moreover, not all driver mutations may result in new immunogenic antigens. A quest for neo-antigen targets does not only require next-generation sequencing techniques to identify tumor-specific mutations (21), but also techniques to determine whether a neo-epitope can be presented by MHC and recognized by T cells (22, 23).

In short, we consider epitopes from selected (non-shared) CTA and neo-antigens as potentially safe T cell target antigens. However, no matter what the antigen, it is recommended to perform stringent *in silico* analysis and preclinical testing to confirm the antigen’s absence from vital organs. Strategies used to identify titin as a cross-recognized peptide, such as amino acid scanning, gene database searches, and use of three-dimensional cell cultures, are potentially helpful in this respect (24). In addition, one could consider using suicide systems to deplete self-reactive T cells prior to proceeding with clinical testing (25–28). Although suicide genes provide the option to delete TCR-transduced T cells, it is questionable whether such a switch could counteract the fast kinetics of toxicity reported in the above-mentioned trials (29, 30).

### Immunogenicity

The immunogenicity of an antigen, i.e., its ability to initiate immune responses, is determined by the level of its expression, how it is processed and presented, and how well it is recognized by T cells.

#### Level of expression and processing of antigens

Ideally, target antigens should be expressed at high levels by most if not all tumor cells. Such a property is generally restricted to those antigens that are related to oncogenesis and that tumors cannot easily do without (see Selective Expression). It is noteworthy that the production of antigens, such as those of MAGE-A family members and NY-ESO1, is enhanced and becomes more homogeneous within tumors by treatment with demethylation agents and/or histone deacetylases (31–34). In a phase II clinical study, in which hematological malignancies were targeted and which included treatments with epigenetic drugs, it was observed that T cell responses directed against CTA were enhanced with no evidence of adverse events (35). In addition, the production of antigens may depend on immune or intermediate proteasomes, rather than standard proteasomes, and on unconventional post-translational events such as reverse splicing and deamidation of proteins (36–38). Such processing of antigens, in particular when mediated by immune proteasomes, may benefit from local production of interferon (IFN)γ. Finally, the release and hence the availability of antigens may be enhanced via treatment-induced cell death following (co-treatments with) chemotherapy, irradiation, and/or therapy with tyrosine kinase inhibitors (39, 40).

#### Cross-presentation of antigens

Antigen cross-presentation may take part in the infiltration of antigen-specific CD8 T cells (41) and cause activation of T cells and subsequent stroma destruction, thereby preventing outgrowth of antigen-negative tumor cells. Recently, Engels and colleagues revealed that peptide:MHC affinities of 10 nM or less allowed for cross-presentation of antigens by stromal cells (42). Notably, using an experimental model in which mice transgenic for TCRs with different antigen specificities were used either as donors or recipients of T cells, they showed that the use of peptide targets that can be cross-presented result in complete anti-tumor responses. Destruction of tumor stroma, a bystander response that may put an advantage to T cells over drugs (43, 44), may require optimal T cell fitness (as measured by production of IFNγ) and IFNγ-mediated preservation of Fas expression by stromal cells (45).

#### Robustness of antigenicity

Loss of tumor antigen expression after infusion of T cells, and its impact on the recurrence of tumors, is an important yet controversial aspect. Decreased antigen expression has been proposed to be a consequence of molecular alterations in tumor cells, such as genetic and epigenetic changes in antigen genes, MHC genes, and genes related to antigen processing and presentation (46–48). Indeed, selective loss of antigen or HLA-A2 expression has been reported in primary and metastatic melanoma lesions in non-treated patients (49, 50) as well as patients treated with T cells (51, 52). Also, Landsberg and colleagues, using a gene-engineered model of melanoma, have eloquently demonstrated that a therapy-resistant phenotype may be directed by an inflammatory milieu and tumor necrosis factor (TNF)α’s ability to lead to epithelial dedifferentiation and decreased expression of melanoma antigens (53). In contrast to these findings, there is increasing evidence to support the view that tumors progress without loss of T cell antigens. In various preclinical models, in which either skin, lung, or ovarium tumors were studied, it was observed that tumors progressed despite continued antigen expression (54–56). In these models, tumor progression was rather a consequence of reduced T cell infiltration and reduced T cell responsiveness. We postulate that in the setting of T cell therapy, loss of target antigen, whether by T cell-dependent selection or epigenetic silencing (57, 58), is *not* necessarily a driving mechanism in tumor recurrence (Straetemans et al., manuscript submitted).

#### Target multiple antigens simultaneously

In current TCR gene therapy trials, single MHC class I-restricted antigens are targeted. Preclinical studies have suggested that the targeting of two or more antigens enhances the therapeutic potential of T cells. For example, adoptive transfer of two CD8 T cell populations to simultaneously target ovalbumin and gp100, rather than either one antigen, resulted in delayed recurrence of tumors (59). Interestingly, treatment with viruses positive for three MHC class II-restricted antigens, i.e., neuroblastoma RAS, TRP1, and cytochrome c1, resulted in complete anti-tumor responses that were accompanied by significant CD4 T helper cell type 17 (Th17) responses (60). Since cooperation of CD4 and CD8 T cells appears important in the effector phase of an anti-tumor response and may contribute to the bystander elimination of tumor stroma (61), it may be worthwhile to simultaneously target MHC class I and II targets. With respect to human antigens, it is interesting to note that X-chromosome linked CTAs are coordinately expressed in tumor tissues (62), which may allow the simultaneous targeting of multiple CTAs.

## Fitness of T cells

The responsiveness of T cells toward tumor antigen is generally tuned down, most likely at various levels. First, reactive T cells may be deleted during T cell development in the thymus; second, peripheral T cells may be susceptibility to anergy; and third, intra-tumoral T cells may require enhanced co-stimulation (63). To overcome such T cell tolerizing mechanisms one can optimize T cell fitness. Here, we define T cell fitness according to the following three T cell properties: functional T cell avidity, T cell co-signaling, and T cell differentiation.

### Functional T cell avidity

Functional T cell avidity is considered as the ability of T cells to respond to a given concentration of cognate peptide antigen, and can be enhanced via strategies, often involving gene-engineering of TCRαβ transgenes, that either increase the level of cell surface expression of TCR chains or the TCR’s affinity for peptide-MHC.

#### Expression level of TCR transgenes

One angle to enhance the surface expression of TCR transgenes is through optimization of the TCR gene transfer methodology, including choice of gene delivery method, use of optimal vector elements, and use of transgene cassettes [reviewed in Ref. (6, 64)]. Another angle to enhance the surface expression of TCR transgenes is through limitation or abolishment of TCR mis-pairing. TCR mis-pairing is the formation of TCR heterodimers that comprise one transgenic TCR chain and one endogenous TCR chain, and represents a phenomenon that is inherent to the generation of TCR-engineered T cells. Importantly, TCR mis-pairing dilutes the surface expression of the transgenic TCRαβ chains, and mis-paired TCRs are of unknown specificity and can yield self-reactive T cells. Although in clinical trials performed so far, no formal observations of toxicities mediated by TCR mis-pairing have been made, preclinical studies have clearly demonstrated that TCR mis-pairing has the potential to induce harmful recognition of self-antigens (65, 66). Strategies to promote preferential pairing between transgenic TCRα and TCRβ chains (and consequently prevent or reduce TCR mis-pairing) can be grouped according to those that depend on gene-engineering of TCR transgenes and those that do not. The first group of strategies are reviewed in Ref. (67). In short, these strategies include murinization of TCR (68), addition of cysteine amino acids to TCR (69, 70), mutations in TCR transmembrane and constant domains (71, 72), and equipment of TCR with a signaling cassette that replaces TCR transmembrane and intracellular domains with the CD3ζ accessory molecule (73, 74). More recently, a limited number of murine amino acids have been identified that are responsible for enhanced expression and preferential pairing of murinized TCRs (75, 76). Similar efforts to minimize the number of amino acids in a CD3ζ signaling cassette failed, and it was observed that properties of TCRs equipped with CD3ζ signaling cassettes are best preserved when incorporating a complete CD3ζ molecule (77). The other group of strategies includes technologies that enhance expression levels of CD3 molecules in T cells and those that interrupt expression of endogenous TCR chains. Co-transfer of CD3 and TCR genes into T cells resulted in higher levels of TCR expression and allowed T cells to respond to lower concentrations of antigen, and to infiltrate and eliminate tumors with faster kinetics (78). RNA interference techniques have been shown to specifically down-regulate the expression of endogenous but not transgenic TCR chains (79, 80). An alternative method encompasses the use of zinc finger nucleases and a sequential knock-out of endogenous TCRα and β chains, followed by introduction and sorting of TCRα and β transgenes (81). The latter method is relatively new and not yet widely or clinically applied, but holds promise to effectively address TCR mis-pairing.

#### Affinity-enhancement of TCRαβ transgenes

Affinity-enhancement of tumor-specific TCRs, and its exploitation, relies on the existence of a window for optimal TCR affinities. The existence of such a window is based on observations that TCRs specific for HLA-A2-restricted pathogens have *K*_D_ values that are generally about 10-fold lower when compared to TCRs specific for HLA-A2-restricted tumor-associated self-antigens (82). In support of this notion are the observations that a high-affinity MART-1/HLA-A2 TCR mediated improved objective response rates compared to a lower affinity MART-1/HLA-A2 TCR, and that an affinity-enhanced NY-ESO1 TCR mediated significant clinical responses (Table 2). Affinity-enhanced TCRs can be obtained through various routes. First, allo-reactive settings can be used to circumvent self-tolerance and yield T cells with a higher avidity when compared to T cells derived from autologous settings (=patients). Examples of such settings include *in vitro* generation of allo-HLA reactive, peptide-specific T cells (83–85), and immunization of mice transgenic for human-MHC or human TCR (86, 87). Second, TCR affinities can be enhanced by rationally designed mutations of the TCR’s complementarity-determining regions (CDRs) (88, 89). Third, high-affinity TCR variants can be selected from a library of CDR mutants by yeast, phage, or T cell display (90–92). Although the affinity of TCRs significantly contributes to the functional avidity of T cells, recent studies warrant caution when therapeutically implementing this strategy. Clinical reports suggest that CDR mutations in TCRs directed against CEA/HLA-A2, MAGE-A3/HLA-A2, and MAGE-A3/HLA-A1, but not NY-ESO/HLA-A2, were possibly related to patient toxicities (Table 2). Investigations whether defined locations and types of mutations are more prone to lead to toxicities than others would most likely benefit further development of CDR-mutated TCRs. Also, preclinical reports suggest the existence of a functional ceiling with respect to TCR affinity (93, 94). In fact, studies with primary human T cells transduced with affinity-enhanced TCRs directed against NY-ESO1/HLA-A2 (93) or gp100/HLA-A2 (Govers et al., manuscript submitted) pointed to the existence of a *K*_D_ threshold of 1–5 μM, below which T cell function became compromised. The functional impairment of high avidity T cells in the presence of high levels of antigen, as is often the case in tumors, may be related to enhanced expression of the exhaustion marker programed cell death (PD1) and enhanced activity of its downstream sarcoma homology domain 2 phosphatase (SHP)1 (95, 96).

### T cell co-signaling

T cell co-signaling is directed by interactions between co-stimulatory or co-inhibitory molecules and their ligands and determines, in addition to interactions between TCR and peptide-MHC, the functional outcome of T cells [reviewed by Chen and Flies (97)]. The best characterized co-stimulatory and co-inhibitory molecules expressed by T cells are CD28 and cytotoxic T-lymphocyte associated protein (CTLA)4, respectively, which both interact with CD80 and CD86 ligands expressed by APCs. More recent examples of co-stimulatory and co-inhibitory molecules include inducible T cell co-stimulation (ICOS), 4-1BB, OX40, CD40, B and T-lymphocyte attenuator (BTLA), and PD1.

Tumors provide continuous stimulation with antigen often in the absence of co-stimulatory ligands, which may result in exhausted T cells with reduced proliferative capacity, reduced effector function (such as IFNγ production) (98), and up-regulated expression of T cell co-inhibitory molecules (99). Immunotherapy with monoclonal antibodies to block the T cell co-inhibitory molecules CTLA4, PD1, PDL1, or the combination of CTLA4 and PD1 showed clear clinical successes in the treatment of advanced melanoma (see Table 1). These clinical activities have provided an impetus for the development of blocking other co-inhibitory molecules and/or stimulation of co-stimulatory molecules (100–104). The beneficial outcome of targeting T cell co-signaling most likely relies on enhancement of infiltration of T effector cells (Teff) into tumor tissue and activation of Teff, as well as depletion of intra-tumoral T regulatory cells (Treg) (103–105). We would advocate explorative studies to test the combination of blocking T cell co-inhibitory molecules and adoptive transfer of Teff. In addition to this combination of immune therapies, two other approaches to implement T cell co-signaling in protocols of T cell therapy have already been clinically tested. First, TCR transgenes can be equipped with a signaling cassette that harbors a co-stimulatory molecule. Such a signaling cassette, designed in analogy to those used in co-stimulatory CARs (6), typically introduces accessory and co-stimulatory molecules to enhance the function of T cells expressing the TCR transgene. It is noteworthy that clinical trials using CARs containing CD28 or CD137 demonstrated significant objective responses in patients with B cell leukemia (106–108), and while CARs may evoke immune responses, these were directed against murine idiotypes, but never against boundaries between genetically introduced human molecules (109). According to this rationale, single and two-chain TCR genes have been coupled to a combination of CD28 and CD3 molecules and were shown to provide T cells with improved function *in vitro* (110, 111) (Govers et al., manuscript submitted). Second, T cells can be stimulated *ex vivo* with human artificial APC (aAPCs) that express co-stimulatory ligands (4, 112). In addition to co-stimulatory ligands, these aAPCs are mostly engineered to express HLA-A × 0201 and used to stimulate T cells in the presence of common-γ cytokines other than interleukin (IL)-2. These combined activations allow for the generation of HLA-A2-restricted, antigen-specific T cells with a less differentiated phenotype (CD45RA^+^ CD62L^+^) and superior T cell functions *in vivo* (112). In a clinical study, T cells educated with aAPC presenting CD80, CD83, and a MART-1 peptide, and cultured in the presence of IL-2 and IL-15, resulted in objective responses in patients with metastatic melanoma (Table 1). Notably, inclusion of T cell co-stimulation by either one of the two above-mentioned approaches relieved the requirement for patient preconditioning with chemotherapy and/or *in vivo* IL-2 administration (4, 106).

### T cell differentiation

The differentiation of naïve T cells into mature CD8 Teff or CD4 Th1 or Th17 cells is required for T cells to make full use of their functional attributes directed against tumor cells, such as cytotoxicity and production of IFNγ and TNFα. The differentiation of T cells is largely driven by environmental stimuli, with cytokines being well-studied examples of such stimuli (113, 114). Progression of T cells into a differentiated subset is not necessarily permanent, and in particular T helper cell subsets have shown plasticity and may change into another T helper cell subset (114). Differentiation of CD8 and CD4 T cells, although occurring according to similar principles, follow different routes and show different outcomes. Strategies to manipulate T cell differentiation to advance T cell therapy are discussed separately for both T cell subsets.

#### CD8 T cells

Naïve CD8 T cells can differentiate, depending on the quantity and quality of the initial antigenic and co-stimulatory stimuli, into stem-cell memory T cells, central memory T cells, effector memory T cells, or T effector cells (115). An important observation that came from preclinical studies was the inverse relationship between CD8 T cell differentiation and proliferation, and hence the inverse relationship between CD8 T cell differentiation and *in vivo* persistence and therapeutic activity (113). Two strategies have been reported to exploit this inverse relationship and improve adoptive T cell therapy. In one such strategy, as shortly mentioned in Section “T Cell Co-Signaling,” T cells are exposed to common-γ cytokines other than IL-2 prior to adoptive T cell transfer. For example, treatments with either IL-7 + IL-15 or IL-15 + IL-21 generated gene-engineered T cells with a less differentiated CD8 T cell phenotype (i.e., central memory phenotype), prolonged peripheral persistence, and potent antigen reactivity (116, 117). In addition to soluble cytokines, Singh and colleagues reported on aAPC that express membrane-bound IL-15 and IL-21 and facilitate the generation of “young” T cells (112). In other reports, the anti-tumor efficacy of T cells was enhanced either via *in vivo* administration of IL-15 + IL-21 (118) or conjugation of nanoparticles, encapsulating these cytokines, to the surface of therapeutic T cells (119). In a second strategy, T cells are enriched for less differentiated T cell populations, i.e., based on CD62L expression, and subsequently used as recipient cells for gene transfer (120, 121). A recently identified population of “stem-cell memory” CD8 T cells, expressing high levels of CD95, IL2Rβ and demonstrating increased proliferative potential and ability to mediate anti-tumor responses, may represent a promising subset of T cells for gene-engineering and therapeutic application (122). In fact, Cieri and colleagues have set up a protocol to obtain and gene-modify stem-cell memory CD8 T cells, which includes the use of CD3/CD28 mAbs and IL-7 and IL-15 and could potentially be translated to a clinical setting (123).

#### CD4 T cells

Naïve CD4 T cells can differentiate into multiple subsets, including Th1, 2, 9, 17, 22, follicular helper and various Tregs, often defined by the expression of “signature cytokines” or typical functions, such as B cell activation or the down-modulation of T cell responses (124). With respect to anti-tumor responses, it appears that upon cell transfer Th1 and Th17 are the most potent CD4 T cell subsets (125, 126). Administration of CD4 T cells, and in particular Th1 cells, has been shown to prevent exhaustion of CD8 T cells, enhance tumor infiltration of CD8 T cells and result in effective tumor eradication (125, 127–130). More recently, it was discovered that adoptive transfer of Th17 cells effectively mediate rejection of TRP1-positive tumors in a TCR-transgenic mouse model (126). Furthermore, Th17 cells appear to be long-lived and their molecular signature resembles that of stem-cell memory CD8 T cells (131). Interestingly, the anti-tumor activity of Th17 cells depended on its (incomplete) differentiation and conversion into Th1 cells, resulting in a co-existence of Th17 and Th1 cells, and it may very well be this multi-potent aspect that provides a therapeutic advantage.

Collectively, these data argue in favor of a combined therapeutic use of CD8 T cells and Th1 or Th17 cells. To this end, CD4 T cells can be functionally endowed with MHC I-restricted TCR and/or CD8 via gene transfer (132–135). Alternatively, one could opt for strategies that induce *in vivo* conversion of CD4 T cells into Th1 cells, such as IL-12, IFNα, IFNγ, or blocking PD1 ligation (136–139). Also, metabolic signals, such as activation of T cell mammalian target of rapamycin (mTOR) and aerobic glycolysis can enhance differentiation toward IFNγ-producing T cells and may be exploited therapeutically (140, 141).

## Sensitization of Micro-Milieu for T Cell Therapy

Tumors, following initial regression upon treatment with T cells, most often become resistant to T cell therapy and recur. Recent understanding suggests that, at least in some tumors, therapy resistance may be part of a negative feedback loop that is initiated once an anti-tumor CD8 T cell has occurred (142). Therapy resistance is often characterized by a dis-balance between numbers and activation state of immune effectors cells versus those of suppressor cells. Strategies to manipulate numbers and activation state of immune cells are discussed separately for effector and suppressor cells.

### Recruitment and activation of immune effector cells

Immune effector cells that have been recognized for their contribution to an anti-tumor response are numerous and, in addition to CD4 and CD8 T cells, include natural killer (NK), natural killer T cells (NKT), macrophages, and neutrophils. Here we will focus on Teff and macrophages and how manipulation of the micro-milieu may enhance their recruitment and activation.

#### Enhance recruitment of T effector cells

Clinical studies have demonstrated an unfavorable prognostic value of a limited CD8 T cell infiltration in melanoma, colorectal and ovarium carcinomas (143–145). Vascular changes have been reported to contribute to arrested T cell infiltration and include insufficient vascular maturation and enhanced expression of endothelin B receptor, regulator of G-protein signaling 5 (Rgs5) and/or extracellular matrix components [reviewed in Ref. (146)]. Such changes may be targeted, as evidenced by angiostatic therapy in which antibodies directed against vascular endothelial growth factor (VEGF) or angiopoietin 2, or in which T cells gene-engineered with a CAR directed against VEGF receptor (VEGFR)2 resulted in enhanced T cell infiltration (147–149). In addition, drugs that inhibit angiogenesis or endothelin receptor B were able to enhance the expression of intercellular adhesion molecule (ICAM)1 on endothelial cells and to normalize T cell infiltration (150, 151). In various solid tumors, T cell infiltration appears to be facilitated by vessels that closely mimic high endothelial venules (HEV) and which may be part of ectopic lymphoid structures in tumor stroma (152, 153). A better understanding of the development of such HEV in tumor stroma may provide novel targets to improve T cell infiltration in tumors.

In addition to vascular changes, spontaneous cutaneous melanoma tumors in mice demonstrated a decreased mRNA expression of chemoattractants that contribute to recruitment of CD8 T cells, such as chemokine (CC motif) ligand (CCL)5 and chemokine (CXC motif) ligands (CXCL)9 and 10 (146). In a subset of patients with melanoma metastases, lack of chemoattractants coincides with limited migration of CD8 T cells and limited presence of lymphoid structures (154). Current findings from our laboratory suggest that a decreased expression of selected chemoattractants and adhesion molecules are related to a decreased infiltration of CD8 T cells and tumor relapse following T cell therapy (Straetemans et. al., manuscript submitted). Interestingly, Hong and colleagues have shown that the chemotherapeutic drugs dacarbazine, temozolomide, and cisplatin enhanced the expression of CCL5, CXCL9, and CXCL10 in patient melanoma, which in turn correlated with improved immune control of tumors (155). Vice versa, T cells when gene-engineered to express chemokine (CXC motif) receptor (CXCR)2 displayed enhanced trafficking toward tumor cells secreting the corresponding chemokine ligand CXCL1 (156). Also, in xenograft tumor models of mesothelioma and neuroblastoma, the genetic introduction of chemokine (CC motif) receptor (CCR)2 in T cells resulted in increased T cell infiltration in tumors secreting CCL2 and was associated with significantly increased anti-tumor activity (157, 158). Other molecules often present in the micro-milieu that, when targeted, resulted in enhanced T cell accumulation at the tumor site are indoleamine 2,3-dioxygenase (IDO) and reactive nitrogen species. Inhibition of IDO by a small molecule blocks tryptophan depletion, enhances T cell infiltration, and delays tumor growth (159). Reactive nitrogen species induce TIL unresponsiveness (160), nitration of the TCR complex (161), and modification of the chemokine CCL2 (162). Drugs affecting the local production of reactive nitrogen species restore TIL function and improve intra-tumoral T cell migration and an anti-tumor T cell response (160, 162). Taken together, the above studies show the drug-ability of molecules that are involved in T cell extravasation and T cell migration into tumor tissues, and advocate studies to combine such drugs with adoptive T cell therapy.

#### Enhance T cell effector functions

Early protocols of adoptive T cell therapy already demonstrated the beneficial effects of co-treatments such as chemotherapy, vaccination, and/or cytokine support on T cell activation [reviewed in Ref. (64)]. More recently, additional strategies that enhance anti-tumor functions of Teff have been reported. A first strategy became apparent from clinical success with additional T cell co-stimulation or blocking of T cell co-inhibition (see T Cell Co-Signaling and Table 1). A second strategy relates to the inhibition of T cell suppressive cytokines, such as transforming growth factor (TGF)β. For example, genetic introduction of a dominant-negative TGFβ receptor II in TCR-engineered T cells resulted in increased anti-tumor T cell responses in a spontaneous tumor model of prostate cancer (163). Another study tested the safety of mouse T cells engineered with this dominant-negative receptor, and could not detect spontaneous proliferation of these T cells *in vivo* (164). Genetic knockdown of negative regulators of T cell activation represents yet another strategy to enhance T cell activation. T cells with siRNA-mediated knockdown of casitas B-lineage lymphoma b (Cbl-b) displayed a lower threshold for T cell activation and, when adoptively transferred in mice with disseminated leukemia, resulted in enhanced anti-tumor effects (165). These latter findings warrant further testing of T cells with enhanced T cell activation, including tests that assess the safe use of these T cells.

#### Enhance recruitment and activation of macrophages

High numbers of macrophages with a tumor-promoting (M2) phenotype, but not those with a tumor-inhibiting (M1) phenotype, correlate with poor prognosis for patients with various cancers (166). When conjugated to a vascular homing peptide and targeted to tumors, TNFα resulted in a switch from M2 to M1 macrophages, which was accompanied by normalization of tumor vasculature and enhanced infiltration of CD8 T cells (167). Interestingly, T cells gene-engineered to release the cytokine IL-12 were shown to improve the therapeutic efficacy of T cells, an effect that is likely mediated by cells of the innate immune system (168, 169). T cells that express IL-12 under the control of the Nuclear Factor of Activated T cell (NFAT) promoter, and deliver IL-12 locally in the tumor environment upon encounter of cognate antigen, induce destruction of antigen-negative cancer cells with a prominent role for monocytes and monocyte-derived TNFα (168). Such findings are not necessarily restricted to IL-12 since IL-15, when provided locally into tumors, also enhanced the responsiveness of adoptively transferred T cells and facilitated the removal of antigen-negative tumor cells (170).

### Reduce numbers and activity of immune suppressor cells

T regulatory cells, M2 macrophages, and myeloid-derived suppressor cells (MDSC) are among the major immune-suppressive cell types in the tumor micro-milieu. Immune suppressor cells can reduce T cell infiltration into the tumor and suppress local T cell responses by: release of reactive nitrogen and oxygen species (171); expression of IDO and arginase (159, 172); and production of cytokines such as TGFβ, IL-4, and IL-13 (173). Despite initial removal of these cells by administration of chemotherapeutic agents, the populations of MDSCs and Tregs may recover at a faster rate than CD4 and CD8 Teff (174). Furthermore, Jensen and colleagues demonstrated that therapeutic CD4^+^ T eff can convert into a Foxp3^+^CD4^+^ Treg population (175). Various strategies have been reported to deplete or inactivate Tregs. These strategies include administration of anti-CD25 antibodies, combined intra-tumoral injection of anti-CTLA4 and OX40 mAbs, or blocking IDO (104, 176). Interestingly, blocking IDO may induce conversion from Treg to Th17 helper cells, which can further contribute to anti-tumor T cell responses (176). With respect to MDSCs, it is of interest to note that classical chemotherapeutic agents, such as docetaxel, are able to deplete these cells. Docetaxel-mediated depletion of MDSC, when combined with adoptive T cell therapy and dendritic cell vaccination, was shown to enhance anti-tumor responses (174). Alternatively, differentiation of MDSC into mature myeloid cells, which can be established upon administration of β-glucans (glucose monomers from cell walls), may also provide an angle to relieve immune suppression (177).

## Future Perspectives

By now, the feasibility of TCR gene therapy studies has been well established by the pioneering trials listed in Figure 1B, and is further enhanced by current optimizations and standardizations of protocols. TCR gene therapy, alike any cell-based therapy, requires specialized good manufacturing practice (GMP) and patient treatment facilities. Such facilities allow the generation and testing of virus batches and the gene processing and expansion of T cells, and are already integrated in multiple academic and private centers. Notably, parameters, such as time-lines and costs to manufacture a therapeutic T cell product, are considered competitive when compared to other clinical-grade products, such as antibodies. An ongoing EU project to treat metastatic esophagus-gastric cancer and melanoma with NY-ESO1 TCR-engineered T cells, in which we participate, shows that time-lines and costs to obtain a T cell product are about 2 weeks and 36 k per patient (13.5 k for production, quality testing, and test runs of virus batch; and 22.5 k for T cell processing), respectively. For comparison: estimated per patients costs of Ipilimumab (3 mg/kg every 3 weeks, 4 times) and Vemurafenib (0.96 g twice daily for 6 months), both registered treatments for metastasized melanoma in The Netherlands since 2012, are 84 and 57 k [Association of Health Insurances (CVZ), The Netherlands]. The next step, and allowing a more valid comparison, would be the testing of T cell therapy versus standard treatment of care in a randomized trial.

Clinical testing of TCR-engineered T cells, when looking at single trials, demonstrated impressive and unprecedented efficacy but at the same time is hampered by treatment-related toxicity and a transient nature of tumor regression (Table 2). There exists a multitude of strategies that are developed and tested toward advanced safety and efficacy of TCR gene therapy. Here, we have defined three challenges and have categorized recent and successful strategies along these three challenges, which have been schematically depicted in Figure 2. With respect to the first challenge, i.e., choice for target antigen, an important criterion is minimal or no expression of such an antigen by healthy tissues. In this respect, non-shared and tumor-restricted CTAs as well as neo-antigens should be considered as potentially safe target antigens. Advances in the isolation and characterization of anti-tumor T cells from individual patient samples may increase the number of CTAs and neo-antigens that may qualify as target antigens. T cell-based recognition of similar, but unrelated peptides should be excluded, and to this end it is strongly recommended to perform stringent *in silico* analysis and preclinical tests to confirm that cross-reactive antigens are absent in healthy tissue. In order to improve patient safety further, measures to allow directed killing of engineered T cells have been tested and should be considered, at least for novel TCRs tested in the near future. In addition to tumor-restricted expression, another criterion to choose target antigens is maximal immunogenicity. Peptide epitopes that are cross-presented or the targeting of a more than a single peptide have been reported to induce complete anti-tumor responses, and may represent examples to consider when selecting target antigens.

With respect to the second and third challenges, i.e., fitness of T cells and sensitization of tumor micro-milieu, we would like to propose a two-step treatment protocol. The first step represents the transfer of fit T cells. T cell fitness involves optimal T cell avidity, additional T cell co-signaling, and using T cells with a preferred differentiation stage. T cell avidity can be optimized by enhancement of TCR affinity, yet reported treatment-related toxicities warrant caution when using affinity-enhanced TCRs (Table 2) and recommend further studies to define rules of TCR binding of cognate versus non-cognate peptides. With respect to T cell co-signaling, antibodies that block T cell co-inhibitory molecules and T cells gene-engineered with co-stimulatory receptors have demonstrated clinical successes. The implementation of such strategies in T cell therapy protocols holds promise for future trials. Also, developments to obtain and gene-modify early differentiation stages of CD8 T cells, including stem-cell memory CD8 T cells, are at the brim of being translated to a clinical setting. Whatever the chosen route, an important measure for T cell fitness *in vivo* is the ability of these cells, whether it be CD8 T cells or certain subsets of CD4 T cells, to produce IFNγ and TNFα. The production of these cytokines not only determines T cell responsiveness, but also to what extent innate immune cells are recruited into the tumor and become activated to further improve an anti-tumor response and potentially avoid tumor relapse. The second step represents antagonism of an immune-suppressed milieu. Various strategies, such as antibodies or drugs to mediate angiostasis, chemotherapeutic agents to enhance intra-tumoral T cell infiltration, and local (T cell-mediated) delivery of cytokines, have proven beneficial to enhance the local ratio between effector and suppressor immune cells. Development of such a two-step protocol, together with the targeting of a selected antigen, is the way forward and expected to further enhance the success rate of TCR gene therapy to treat solid tumors.

## Conflict of Interest Statement

The authors declare that the research was conducted in the absence of any commercial or financial relationships that could be construed as a potential conflict of interest.

## Footnotes

^1^www.clinicaltrials.gov

^2^http://www.cta.lncc.br
